# Pathogenesis of Hazara orthonairovirus infection in type I interferon receptor-deficient mice and resolution of disease following 4′-fluorouridine therapy

**DOI:** 10.1128/jvi.01421-25

**Published:** 2025-11-18

**Authors:** Justin S. Murray, Jonna B. Westover, Dionna Scharton, Arnaud J. Van Wettere, Alexander A. Kolykhalov, Shuli Mao, Michael G. Natchus, George R. Painter, Brian B. Gowen

**Affiliations:** 1 Department of Animal, Dairy and Veterinary Sciences, Utah State University 228472 , Logan, Utah, USA; 2 Department of Veterinary and Clinical Life Sciences, Utah State University 4606, Logan, Utah, USA; 3 Utah Veterinary Diagnostic Laboratory, Logan, Utah, USA; 4 Emory Institute for Drug Development, Emory University 1371, Atlanta, Georgia, USA; 5 Drug Innovation Ventures at Emory (DRIVE), Atlanta, Georgia, USA; 6 Department of Pharmacology and Chemical Biology, Emory University 197252, Atlanta, Georgia, USA; University of Minnesota Twin Cities, Minneapolis, Minnesota, USA

**Keywords:** Hazara virus, animal models, broad-spectrum antiviral, nairovirus, Crimean-Congo hemorrhagic fever virus

## Abstract

The Crimean-Congo hemorrhagic fever orthonairovirus (CCHFV) is a widespread tick-borne agent that causes severe disease in humans. Its expanding geographic range poses a significant public health threat. Although animal models for CCHFV infection have been developed, the requirement for maximum biosafety level 4 facilities limits the development of countermeasures against the virus. Hazara virus (HAZV) is a closely related orthonairovirus that can be handled in biosafety level 2 containment and has been proposed as a prototype virus for the *Orthonairovirus* genus to facilitate early-stage countermeasure development against the highly pathogenic CCHFV. Here, we present a detailed characterization of HAZV infection in mice deficient in type I interferon signaling (*Ifnar^−/−^*), providing insights into the natural history of orthonairovirus disease and highlighting similarities and differences between the HAZV and previously described CCHFV mouse infection models. We then utilized the HAZV mouse infection model to test the efficacy of a promising broad-spectrum antiviral, 4′-fluorouridine (4′-FlU). Our findings demonstrate that delayed intervention with orally administered 4′-FlU can rescue clinically ill mice following challenge with a lethal dose of HAZV, supporting further investigations of the compound’s efficacy in CCHF disease models.

**IMPORTANCE:**

The Crimean-Congo hemorrhagic fever orthonairovirus poses a significant public health threat, underscored by the expansion of *Hyalomma* genus tick vectors and the lack of clinically proven therapeutic options. The related Hazara orthonairovirus (HAZV), which has not been reported to cause human disease, has been proposed as a prototype virus for the *Nairoviridae* family. Here, we characterize in detail the mouse model of lethal HAZV disease to gain further insight into nairovirus pathogenesis and use the model for the preclinical development of a promising broad-spectrum antiviral drug candidate, 4′-fluorouridine (4′-FlU). Our findings highlight the value of HAZV as a surrogate for proof-of-concept studies supporting early-stage antiviral drug studies and the therapeutic potential of 4′-FlU for the treatment of often-fatal Crimean-Congo hemorrhagic fever.

## INTRODUCTION

Crimean-Congo hemorrhagic fever virus (CCHFV) is a widespread tick-borne virus that can cause severe hemorrhagic disease in humans known as Crimean-Congo hemorrhagic fever (CCHF). The virus belongs to the *Orthonairovirus* genus, in the *Nairoviridae* family, *Hareavirales* order, *Bunyaviricetes* class, and cases of CCHF have been reported throughout Southern Europe, the Middle East, Asia, and Africa (1, 2). The geographic range of CCHFV mirrors that of its *Hyalomma* tick genus vectors. The expansion of *Hyalomma* ticks heightens concerns of the further spread of CCHFV into new areas, broadening the public health threat (3–5). The World Health Organization has classified CCHFV as a high-priority pathogen since 2015 due to case fatality rates of 30% or higher and a lack of clinically proven therapeutic options (4, 6).

Animal models emulating CCHFV infection and disease are vital to advancing our knowledge of CCHFV pathogenesis and developing preventative and therapeutic countermeasures. Several mouse and non-human primate models infected with different strains of CCHFV have been well characterized (7–10). However, work with the virus requires maximum biosafety level 4 (BSL-4) containment facilities, which are not readily available to most researchers. Toward addressing this limitation, the closely related Hazara orthonairovirus (HAZV), which has not been reported to cause human disease, has been proposed as a prototype virus for the *Nairoviridae* family and surrogate for proof-of-concept studies supporting early stages of antiviral drug development targeting CCHFV (11, 12).

Previously, HAZV has been shown to cause severe disease in type I interferon receptor-deficient (*Ifnar^−/−^*) mice (12). While this work established the *Ifnar^−/−^* mouse model, the lack of a more comprehensive assessment of the pathogenesis and host response to infection limited the comparison to CCHFV murine models of disease. To address this gap, we studied the course of HAZV infection and disease progression in *Ifnar^−/−^* mice, including temporal analysis of virologic, hematologic, biochemical, clinical, and immunological parameters. Furthermore, we used the model to investigate the anti-orthonairoviral activity of a promising ribonucleoside analog, 4′-fluorouridine (4′-FlU), which has been reported to be effective against multiple RNA viruses, including CCHFV and other members of the *Bunyaviricetes* class (13–17).

## RESULTS

### HAZV replicates systemically to high titers and causes a profound inflammatory immune response during clinically apparent disease in *Ifnar*^−/−^ mice

**Fig 1:**
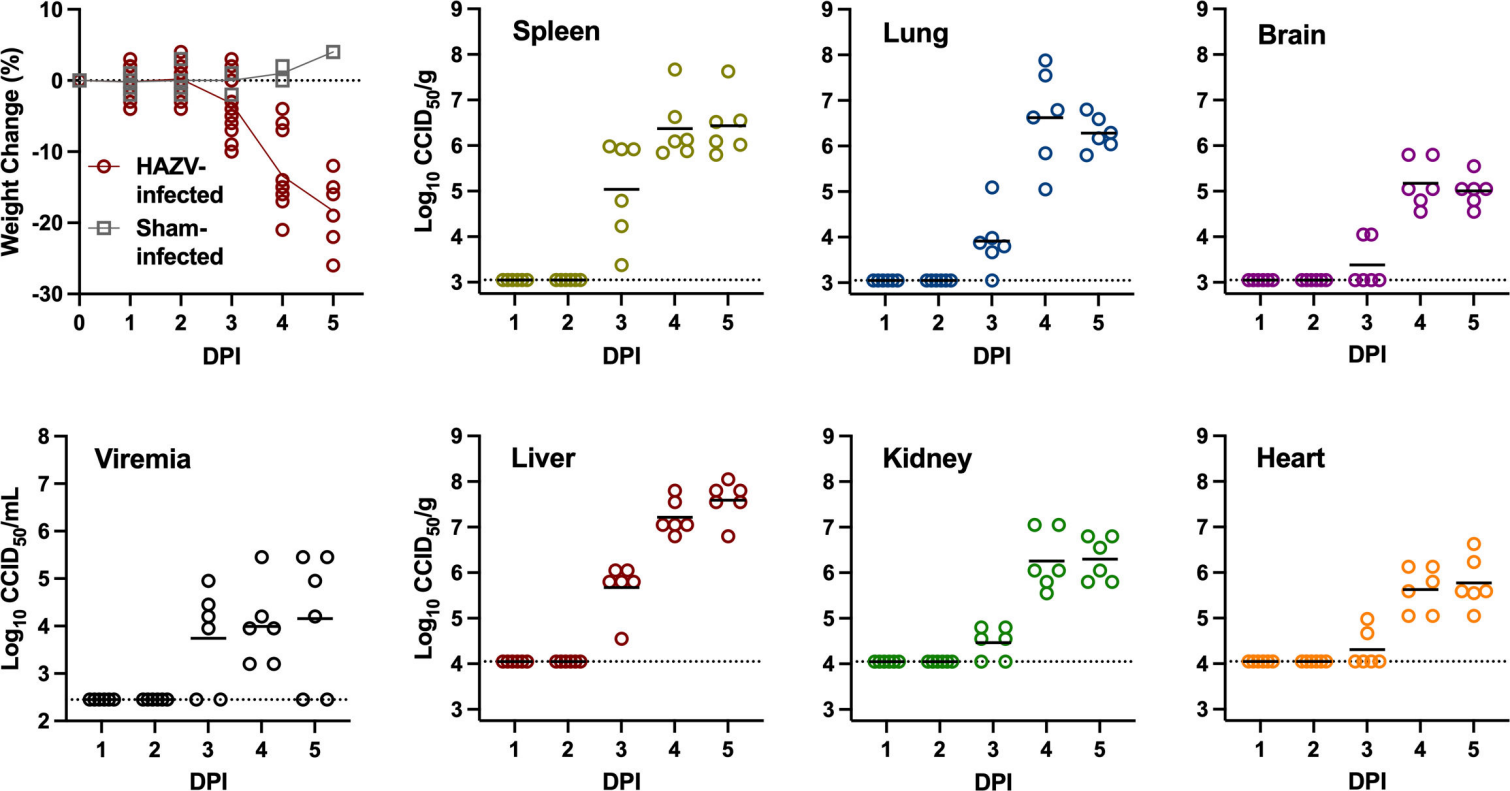
Percent weight change and virus titers in *Ifnar^−/−^* mice challenged with HAZV. Animals were weighed before challenge and randomly assigned to groups for sacrifice on days 1–5. The data represent the group means and standard error of the percent change in weight of surviving animals relative to their starting weights on the day of challenge. Sham-infected normal controls sacrificed on days 1–5 (one animal per day) were included for comparison. When animals were sacrificed on the specified days post-infection, serum, liver, spleen, kidney, heart, lung, and brain were collected for analysis of virus titers. The lower limit of detection (LLD) was 2.45 log_10_ CCID_50_/mL for serum, 3.05 log_10_ CCID_50_/g for spleen, lung, and brain tissue, and 4.05 log_10_ CCID_50_/g for liver, kidney, and heart tissue. The dotted line defines the LLD. DPI, day post-infection.

To more comprehensively characterize HAZV infection and disease in mice, the natural history of disease and host response at selected time points during the acute infection in 6- to 8-week-old *Ifnar^−/−^* mice was evaluated. The HAZV challenge dose selected for the study was 100 CCID_50_ (median cell culture infectious dose), which is approximately 25 × LD_50_ (median lethal dose) of the virus stock. Mice were challenged subcutaneously (SC) with the 100 CCID_50_ dose of HAZV, and groups of animals were sacrificed for sample collection and downstream analyses on days 1–5 post-infection (p.i). Substantial weight loss was observed in infected mice compared to sham-infected normal control animals beginning day 3 p.i. and continued until the final group was sacrificed on day 5 p.i. (Fig. 1). Starting on day 4 p.i., all HAZV-infected mice appeared lethargic, with hunched posture and ruffled fur. Low concentrations of virus were first detected in the serum and tissues of most animals by day 3 p.i. By day 4, infectious virus was detectable in all serum and tissue samples, with the highest viral burdens present in liver tissue (Fig. 1).

**Fig 2:**
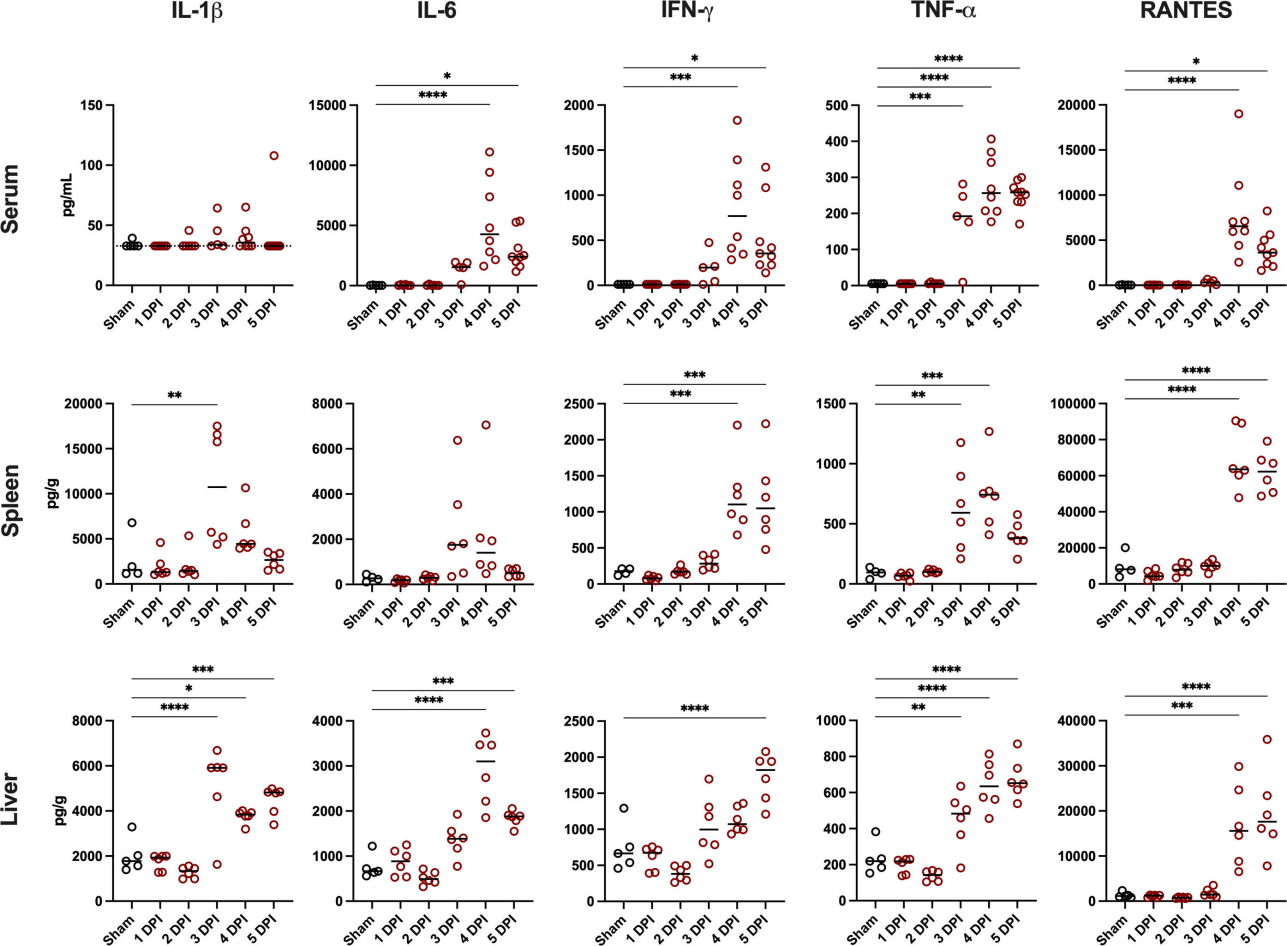
Cytokine response to HAZV infection in *Ifnar^−/−^* mice. Data from six infected mice per day and five sham-infected normal control animals (except for one spleen sample from a sham animal). All serum and tissue values are expressed as pg/mL and pg/g, respectively. ****P < 0.0001, ***P < 0.001, **P < 0.01, *P < 0.05 compared to sham-infected controls (*n* = 5 for serum and liver; *n* = 4 for spleen). A horizontal line representative of the lower limit of detection for serum IL-β concentrations is displayed.

Aligning with the onset of clinical disease and viral replication, concentrations of IL-1α, IL-1β, TNF-α, and MIP-1α were significantly increased in the spleen and liver by day 3 p.i. (Fig. 2; Table S1). In the serum, significantly elevated TNF-α concentrations were also detected by day 3 p.i. Additionally, RANTES was increased dramatically by day 4 p.i. in the serum, spleen, and liver. Furthermore, proinflammatory cytokines, such as IL-6 and IFN-γ, were significantly elevated in the serum and liver, and the serum and spleen, respectively, by day 4 p.i. Concentrations of the anti-inflammatory cytokine, IL-10, spiked in the serum on day 4 p.i. and doubled in the liver by day 5 (Table S1). Notably, all 15 cytokines measured were significantly increased in the liver on day 5 p.i. (Fig. 2; Table S1).

### Clinical pathology of *Ifnar*^−/−^ mice infected with HAZV

**Fig 3:**
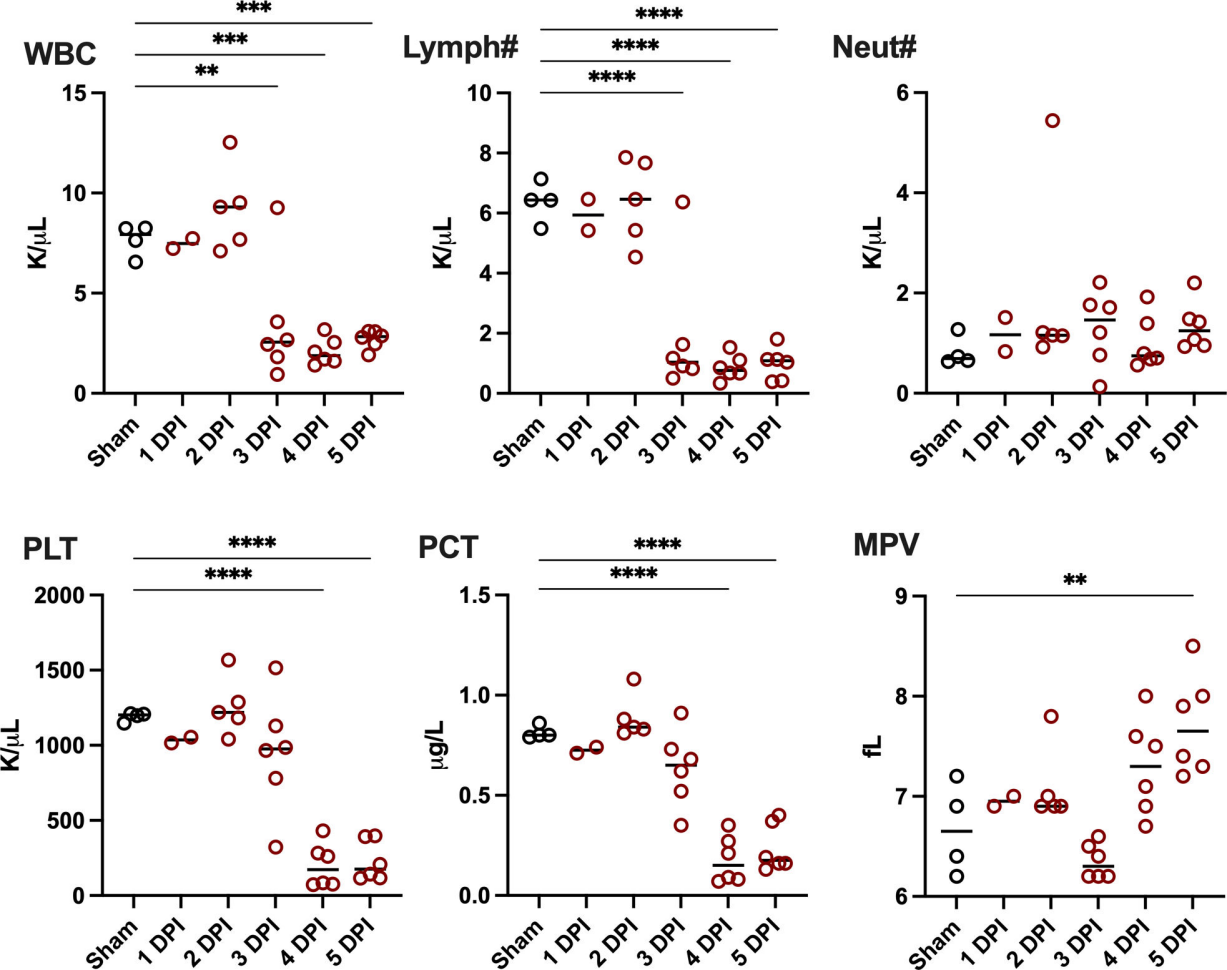
Complete blood cell counts of HAZV-infected mice. Six virus-challenged animals and one sham-infected animal were sacrificed daily on days 1–5 p.i. Blood samples obtained from four animals in the day 1 group, one from day 2, and one of the control animals were inadequate for CBC analysis. ****P < 0.0001, ***P < 0.001, **P < 0.01, *P < 0.05 compared to normal controls (*n* = 4). WBC, white blood cells; Lymph#, lymphocytes; Neut#, neutrophils; PLT, platelets; PCT, plateletcrit; MPV, mean platelet volume.

To investigate the effects of HAZV infection on hematological parameters of *Ifnar^−/−^* mice, anticoagulated whole blood samples were collected, and complete blood count (CBC) was assessed. A marked decrease in the number of lymphocytes, possibly due to lymphocyte necrosis, was observed starting 3 days after virus challenge (Fig. 3). The total white blood cell (WBC) count was also impacted, with profound leukopenia starting on day 3 p.i.; meanwhile, no change in the number of neutrophils was observed. A significant decrease in platelets (PLT) and plateletcrit (PCT) was evident, indicating thrombocytopenia, and the mean platelet volume (MPV) increased on day 5, indicative of platelet activation (Fig. 3). Significant changes in platelet distribution width and erythrocytic parameters were not observed (Table S2).

**Fig 4:**
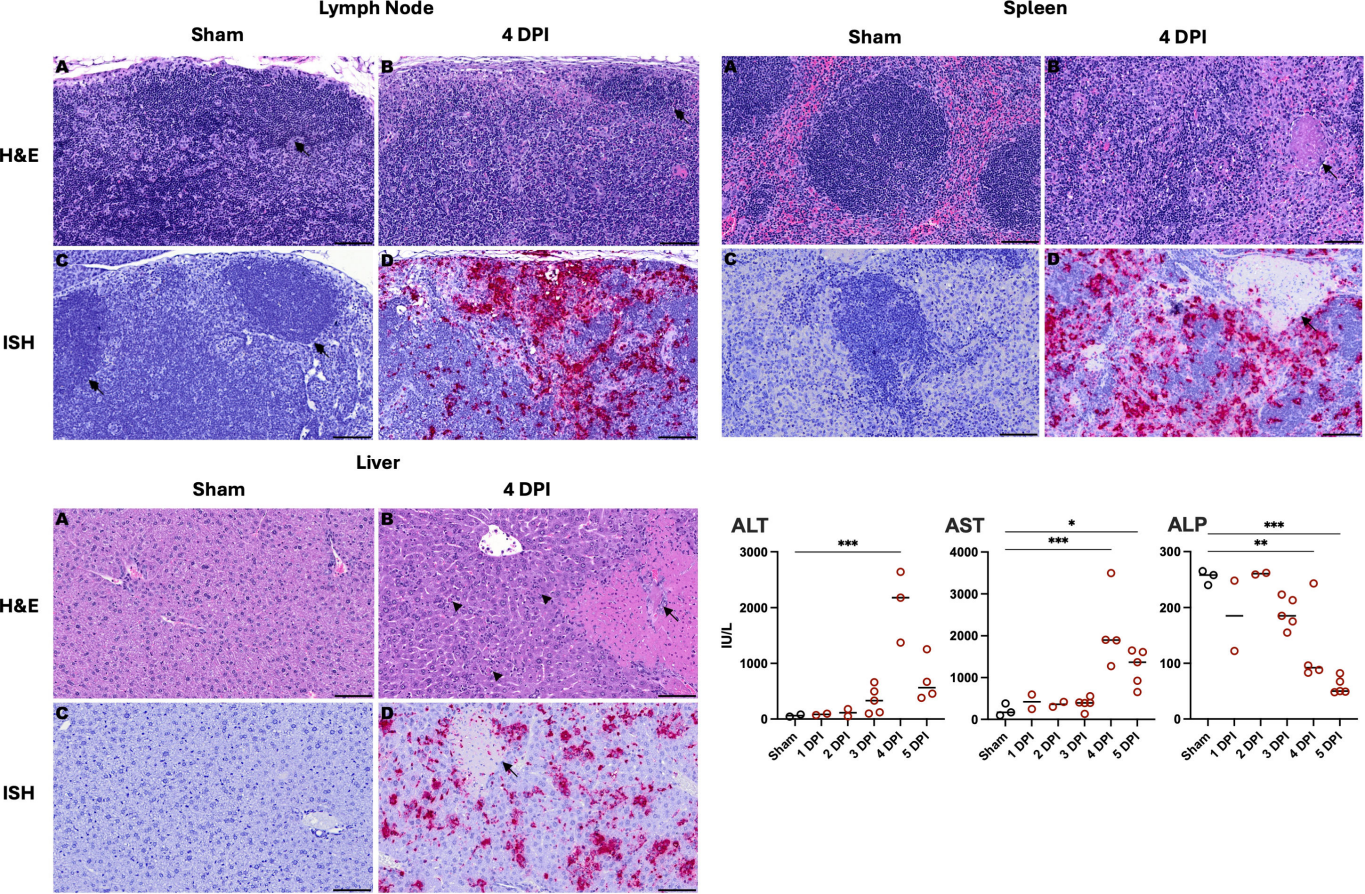
Histology, HAZV RNA *in situ* hybridization, and serum ALT, AST, and ALP concentrations of *Ifnar^−/−^* mice infected with HAZV. Lymph node (top left), spleen (top right), and liver (bottom left) panels are shown. (**A and B**) Histopathology hematoxylin and eosin (H&E) stain subpanels and (**C and D**) *in situ* hybridization (ISH) with hematoxylin counterstain subpanels. Lymph node: (**A**) sham-infected mouse (normal B cell follicle, arrow), (**B**) diffuse lymphoid necrosis (depleted B cell follicle, arrow) and neutrophilic inflammation in an infected mouse at day 4 p.i., (**C**) sham-infected mouse with no viral RNA detected (normal B cell follicle, arrow), and (**D**) viral RNA present in cells with a morphology consistent with endothelial cells and macrophages and lymphocytes in an infected mouse at day 4 p.i. Spleen: (**A**) sham-infected mouse, (**B**) lymphoid necrosis, neutrophilic inflammation, and thrombosis (arrow) in an infected mouse at day 4 p.i., (**C**) sham-infected mouse with no viral RNA detected, and (**D**) viral RNA is present in cells with a morphology consistent with endothelial cells and macrophages and lymphocytes in an infected mouse at day 4 p.i. Liver: (**A**) sham-infected mouse, (**B**) individual hepatocyte necrosis (arrowheads), neutrophilic inflammation, and thrombosis (arrow) surrounded by coagulative necrosis in an infected mouse at day 4 p.i., (**C**) sham-infected mouse with no viral RNA detected, and (**D**) viral RNA present in cells with a morphology consistent with hepatocytes and endothelial cells in an infected mouse at day 4 p.i. Viral RNA is also detected in presumed blood cells within a thrombus (arrow) occluding a portal vein. 100× magnification. Bar = 100 µm. A marked increase in (lower right panel) serum ALT and AST, and a decrease in ALP are observed in infected mice at days 4 and 5 p.i. Data are reported as the group mean and standard deviation. ***P < 0.001, **P < 0.01, *P < 0.05 compared to sham-infected normal controls.

Serum alanine transaminase (ALT), aspartate transaminase (AST), and alkaline phosphatase (ALP) were measured to assess hepatic injury. Adequate amounts of serum were not available for all liver biomarkers for all mice, but measurements were made in at least three mice at days 4 and 5 p.i. We observed that ALT and AST concentrations increased as the disease progressed in the *Ifnar^−/−^* mice between days 2 and 5 p.i. (Fig. 4, bottom right). Interestingly, we found decreasing ALP levels at 4 to 5 days p.i. The cause of decreased ALP is unclear, but reduced concentration of ALP is not considered clinically relevant. Decreases in serum ALP have been observed in rats following hypophysectomy and after fasting (18, 19).

**Fig 5:**
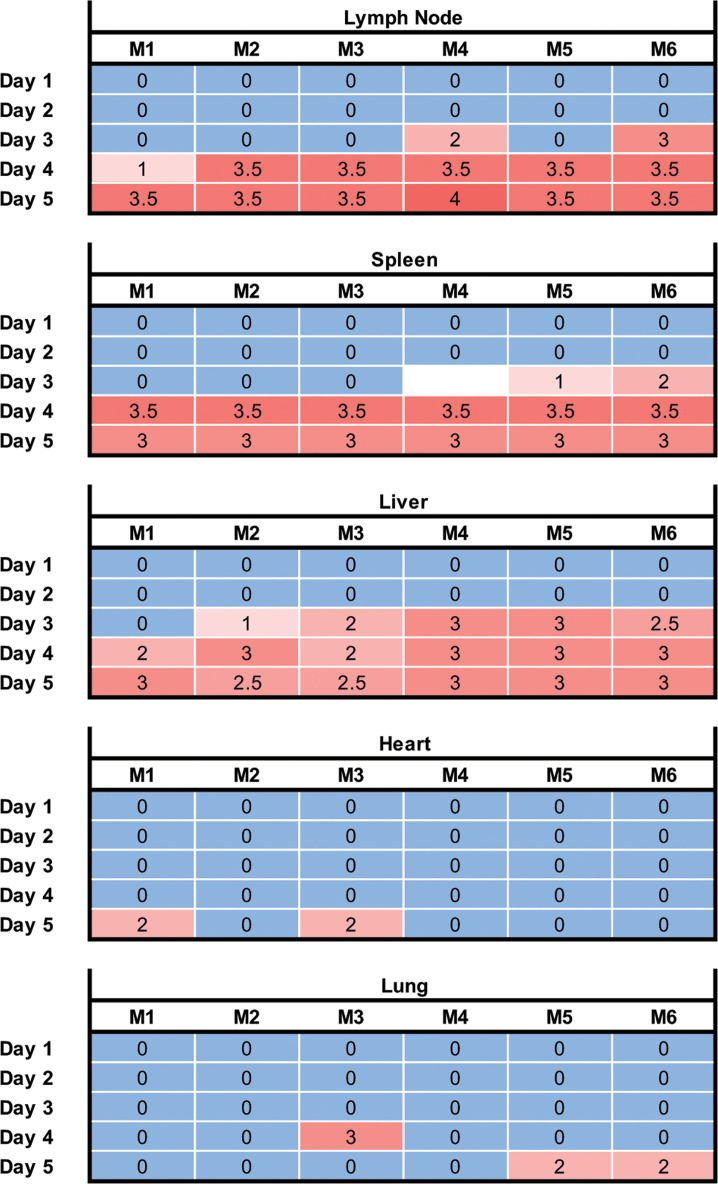
Histologic lesions severity summary of mice challenged with HAZV represented as a heat map. Tissue lesions for each mouse were scored as 0 = absent, 1 = minimal, 2 = mild, 3 = moderate, or 4 = severe. The lesions in the lymph node, spleen, heart, and liver are inflammatory lesions with necrosis and thrombosis. The lesions in the lung are limited to thrombosis with no associated necrosis or inflammation. The spleen from one of the six mice sacrificed on day 3 p.i. was compromised and, therefore, could not be analyzed (no score). No lesions were found in the trachea, esophagus, small intestine, thymus, salivary glands, thyroid gland, adrenal gland, kidney, or brain.

Histopathology lesions and severity correlated well with the hematology and serum chemistry data (Fig. 4 and 5). Microscopic lesions consisted of necrosis and neutrophilic inflammation in lymph nodes, spleen, and liver starting on day 3 p.i. and increasing in severity on days 4 and 5 p.i. (Fig. 5). Vasculitis and thrombosis were also present in lymph nodes, spleen, liver, and lungs (Fig. 4, lymph node panel B, spleen panel B, and liver panel B; Fig. S1J). Lymph node and splenic lesions consisted of multifocal to coalescing lymphoid necrosis with infiltration of neutrophils, histiocytes, and multifocal thrombosis in the white and red pulps (Fig. 4, lymph node panel B and spleen panel B). Two patterns of hepatocellular necrosis were present. The first pattern consisted of multifocal individual hepatocellular necrosis on day 3 p.i., progressing toward multifocal to coalescing small areas of lytic hepatic necrosis on days 4 and 5 p.i. (Fig. 4, liver panel B). This pattern of multifocal lytic necrosis was due to infection of hepatocytes, as detected by *in situ* hybridization (ISH; Fig. 4, liver panel D). In addition, multifocal areas of coagulative midzonal to panlobular necrosis adjacent to thrombosed vessels in portal tracts were scattered randomly in the liver but seemed to occur more frequently in subcapsular hepatic lobules. Little to no virus was detected by ISH in these areas of coagulative necrosis or within thrombi (Fig. 4, liver panels B and D). Pulmonary lesions consisted of thrombosed vessels not associated with inflammation, necrosis, or pneumonia (Fig. S1J). The neutrophilic inflammation in the heart in two out of six animals at day 5 p.i. was focal, involving only a small area, and therefore classified as mild myocarditis and epicarditis (Fig. S1F). No significant microscopic lesions were found in the brain, salivary glands, thyroid gland, trachea, esophagus, thymus, pancreas, small intestine, adrenal gland, or kidney.

The distribution of HAZV GPC precursor viral RNA across multiple organs was evaluated in tissue sections from animals sacrificed 4 days p.i. via ISH (RNAscope). Staining indicating virus replication was present within hepatocytes, endothelial cells in all organs evaluated, adrenal cortical cells, lymphocytes in lymph nodes and spleen, cells with a morphology consistent with monocytes in blood vessel lumens and macrophages in lymph nodes and spleen, and possibly in Kupffer cells in the liver (Fig. 4, lymph node panel D, spleen panel D, and liver panel D; Fig. S1D, H, L, and P).

### Treatment of HAZV infection with 4′-fluorouridine

4′-FlU is a promising ribonucleoside analog shown to have low-µM antiviral activity in cell culture against a CCHFV (16). Here, the compound was first evaluated against HAZV infection in Vero E6 cells cultured in media containing increasing doses of non-toxic concentrations of 4′-FlU or favipiravir for 4 days, at which time the culture supernatants were collected, and their infectious virus yields were determined. Against HAZV, the 90% effective concentration (EC_90_) of 4′-FlU was 4.74 ± 0.59 µM, and the 50% cytotoxic concentration (CC_50_) was >100 µM. The EC_90_ of favipiravir was 10.06 ± 1.02 µM, and the CC_50_ was >500 µM.

Having demonstrated low µM antiviral activity in cell culture, the efficacy of 4′-FlU was evaluated in the HAZV *Ifnar^−/−^* mouse infection model. The compound was administered per os (PO), once daily (QD) for 8 days in 6- to 9-week-old *Ifnar^−/−^* mice challenged with a dose of approximately 20 CCID_50_ of HAZV. Complete and significant protection (*P* < 0.01) compared to the vehicle placebo group was observed in the cohorts treated with 15 or 5 mg/kg 4′-FlU or 200 mg/kg/day favipiravir; the latter dosed twice daily by intraperitoneal (IP) injection (Fig. S2A). Only one animal succumbed to the virus infection in the low-dose 1.5 mg/kg 4′-FlU group. Limited weight loss was observed in drug-treated mice during the first few days of treatment before they stabilized and began to recover lost weight (Fig. S2B). On day 6 p.i., weight loss was significantly more prominent in mice treated with the vehicle placebo compared to the groups treated with 4′-FlU or favipiravir (Fig. S2C). HAZV was not detected in the serum, liver, or spleen of mice treated with 4′-FlU or favipiravir (Fig. S2D).

**Fig 6:**
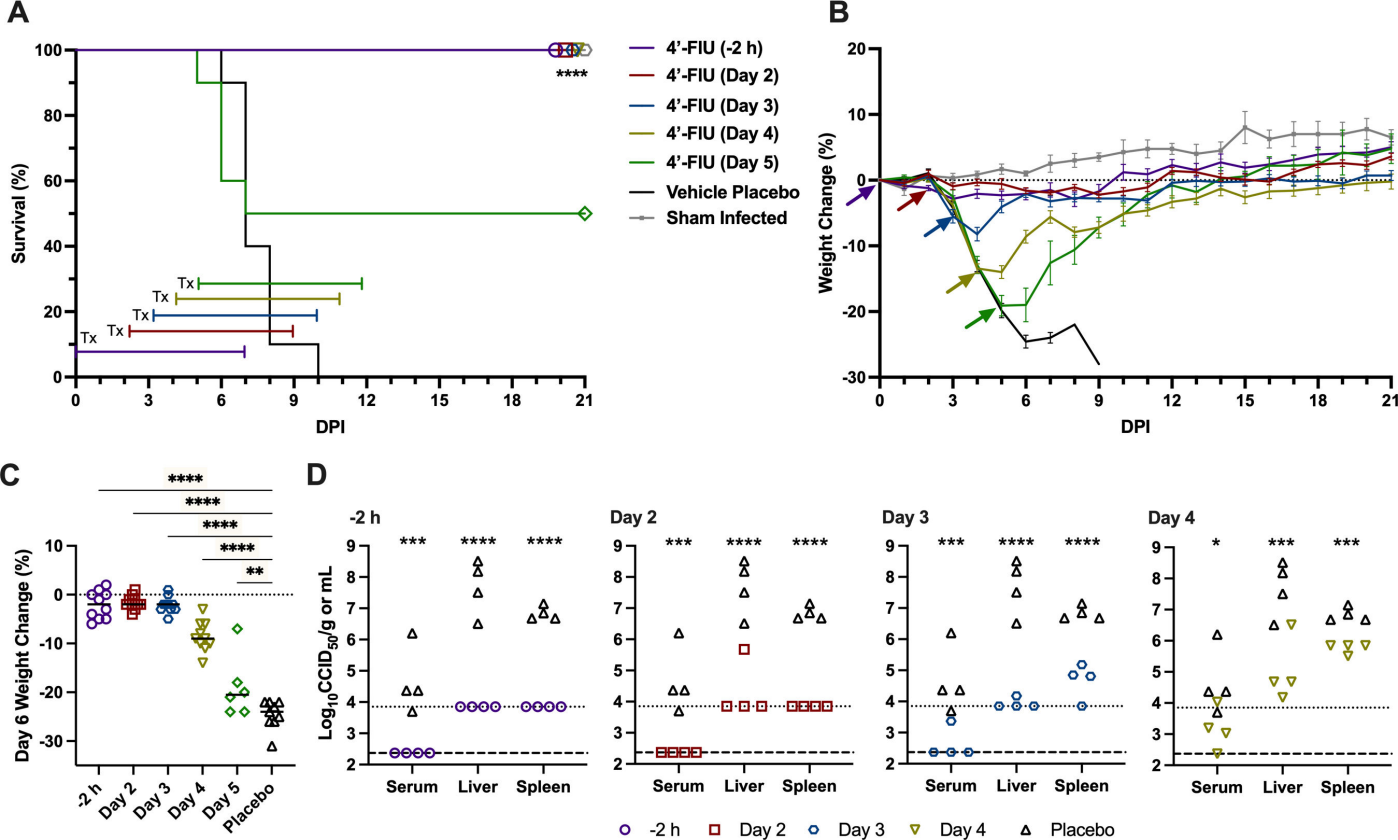
Effect of 4′-FlU treatment on survival, weight loss, viremia, and tissue titers of *Ifnar^−/−^* mice challenged with HAZV. Animals were challenged with 100 CCID_50_ of HAZV and treated PO with 10 mg/kg of 4′-FlU (*n* = 10/group) at the indicated time p.i. Sham-infected normal controls (*n* = 4/group) were included for comparison. (**A**) Survival outcome and (B) daily body weight data represented as the group mean and standard error (SEM) of the percent change in weight of mice relative to their starting weights on day 0, the day of virus challenge. Respective, color-coded treatment windows (**A**) and arrows indicating the time point at which treatments began (**B**) are shown. (**C**) Percent weight change of surviving animals on day 6 p.i. The horizontal lines indicate the group means. (**D**) Subsets of animals in each group (*n* = 4) were predesignated for sacrifice on day 5 p.i. to assess viremia and liver and spleen virus titers. The dashed and dotted lines indicate the assay’s lower limit of detection. *P < 0.05, **P < 0.01, ***P < 0.001, ****P < 0.0001, compared to vehicle placebo control animals. DPI, day p.i.

To test the therapeutic efficacy of 4′-FlU, 6- to 8-week-old *Ifnar^−/−^* mice were challenged with 100 CCID_50_ of HAZV and QD treatments administered PO were initiated 2, 3, 4, or 5 days p.i., with days 4 and 5 representing times when the mice are experiencing considerable weight loss (Fig. 1). Highly significant and complete protection (*P* < 0.0001) was observed in the experimental groups where 4′-FlU treatments were initiated on or before day 4 p.i. (Fig. 6A). Partial protection was observed in the group where treatment began on day 5 p.i. The virus challenge was fully lethal (100%) in the placebo group and, as expected, starting treatment 2 h before infection (positive control group) conferred complete protection (Fig. 6A).

Consistent with the dramatic weight loss of approximately 15% in the day-4 treatment initiation group (Fig. 6B), mice were visibly ill (lethargic, ruffled fur, hunched posture) when they received their first oral dose of 4′-FlU. Remarkably, the animals stabilized and began gaining weight within 24–48 h after their first treatment on day 4 p.i. In the day 5 treatment initiation group, mice had lost approximately 20% of their starting body weight before the first treatment (Fig. 6B), with one animal in the group succumbing to HAZV infection before it could be dosed (Fig. 6A). Despite the advanced disease presentation in this group, the 4′-FlU treatment rescued 50% of the mice (Fig. 6A). On day 6 p.i., weight loss was more prominent in mice treated with the vehicle placebo compared to the groups treated with 4′-FlU starting within 4 days of the HAZV challenge (Fig. 6C). This panel also shows that mice that lost 18%–24% of their starting body weight ultimately recovered from the severe infection and disease.

The impact of the 4′-FlU treatments on infectious viral loads was also measured in subsets of mice sacrificed on day 5 p.i. In the serum, liver, and spleen, HAZV titers were significantly and dramatically reduced in mice dosed with 4′-FlU compared to placebo-treated mice in a treatment time initiation-dependent manner (Fig. 6D). The virus was undetectable in the positive control 2 h pre-treatment group and mostly absent in mice treated starting on day 2 p.i.

## DISCUSSION

In this study, we have more comprehensively characterized the pathogenesis of HAZV infection in *Ifnar^−/−^* mice and utilized the model to test the therapeutic efficacy of 4′-FlU against orthonairovirus infection. This report highlights the utility of the HAZV *Ifnar^−/−^* mouse model as a prototype for developing countermeasures to treat CCHFV infection and disease (11). During our investigation, we observed systemic virus replication with high loads of infectious virus detected in the liver and spleen, similar to what has been reported in characterized CCHFV small animal models (7, 8, 20). Additionally, we reported an increase in proinflammatory cytokines in the serum, liver, and spleen following time points of increased HAZV replication. Similar increases in serum IL-6, TNF-α, MIP-1α, RANTES, and IFN-γ proinflammatory cytokines have been reported for CCHFV-infected mice during advanced disease (7–9, 21). Likewise, *Ifnar^−/−^* mice infected with HAZV share elevated IL-6, IL-10, and TNF-α profiles with humans that experience severe CCHF disease (22, 23). Hamsters deficient in signal transducer and activator of transcription 2 (*STAT2^−/−^*) similarly have an increase in mRNA expression of the proinflammatory cytokines TNF-α and INF-γ following challenge with CCHFV (20). Furthermore, cynomolgus macaques infected with CCHFV strains Hoti and Afg09 reportedly have increased serum IL-6, IL-10, and IL-17A (10, 24, 25). In contrast, *Ifnar^−/−^* mice infected with HAZV did not present with elevated serum IL-17A, instead having significantly increased liver IL-17A 5 days p.i.

Thrombocytopenia and leukopenia are commonly observed in human cases of CCHF (26, 27). Consistent with observations made during the characterization of CCHFV infection in *Ifnar^−/−^* mice, we observed a significant decrease in platelets and lymphocytes in HAZV-infected mice (9). Total WBC counts were impacted during HAZV infection, with profound leukopenia due to lymphopenia observed starting on day 3 p.i., similar to that observed in *STAT1*^*−/−*^ mice infected with CCHFV (8). Our data, as well as that of other CCHFV-infection models in mice, differ from what was reported in *STAT2^−/−^* hamsters infected with CCHFV. The latter showed increases in platelet, lymphocyte, monocyte, neutrophil, and total WBC counts before the animals succumbed to infection (20). Lymphopenia and neutropenia have been documented in cynomolgus macaques infected with CCHFV (10, 24, 25). However, we did not observe significant changes in the number of neutrophils in HAZV-infected mice.

During our investigation on HAZV histopathology, we observed severe lesions in secondary lymphoid tissues and the liver comparable to what has been reported in the HAZV mouse model and CCHFV mouse and hamster models (7–9, 12, 20). Multifocal random lytic necrosis and inflammation associated with the presence of virus (as determined by ISH) were present as expected, with viral infection significantly affecting the liver. Interestingly, midzonal to panlobular coagulative necrosis with often visible portal vein thrombosis in the adjacent portal tract was also present. None-to-minimal amounts of viral RNA were detected in the areas of coagulative necrosis, suggesting that necrosis was likely secondary to thrombosis and ischemia. Thrombosis and associated midzonal to panlobular hepatic necrosis have been reported in wild-type C57BL/6 mice infected with CCHFV but not *STAT1^−/−^* or *Ifnar^−/−^* immunocompromised mouse infection models, immunocompetent mice infected with mouse-adapted (MA)-CCHFV, or the *STAT2^−/−^* hamster CCHFV-infection model (7–9, 20, 28). Localized HAZV replication detected by ISH staining was similar to that observed with MA-CCHFV localization of viral antigen found within hepatocytes, endothelial cells, and cells with a morphology consistent with macrophages in lymph nodes and spleen, and in Kupffer cells in the liver (7). Similarly, a recombinant reporter CCHFV was shown to have tropism for the liver, spleen, adrenal gland, heart, lungs, Peyer’s patches, and reproductive organs in *Ifnar^−/−^* mice (29). Furthermore, immunohistochemical (IHC) and ISH localization of CCHFV in cynomolgus macaques was observed in hepatocytes, Kupffer cells, lymphocytes in the lymph nodes and spleen, endothelial cells of the liver, kidney, and adrenal gland, and in medullary and cortical cells of the adrenal gland (10). Localization of CCHFV via ISH and IHC in humans was reported in hepatocytes, Kupffer cells, endothelial cells, and mononuclear phagocytes (30).

ALT and AST markers were elevated as the disease progressed in *Ifnar^−/−^* mice challenged with HAZV. This finding correlates with the hepatic necrosis observed microscopically and is similar to what has been observed in CCHFV-infected mice (7–9). Likewise, in *STAT2^−/−^* hamsters, elevated ALT concentrations were observed during early time points and increased ALP concentrations during later time points of CCHF infection (20). Fatal outcomes in human CCHF patients have been associated with elevated levels of ALT and AST (27, 31, 32). Elevated concentrations of these liver enzymes have also been observed in non-human primates infected with CCHFV (10, 25). Although uncertain as to why, we observed decreased ALP concentrations 4 days p.i. during time points at which HAZV-infected mice exhibit clinical signs of disease. Patients infected with CCHFV are more commonly reported to have increased concentrations of ALP, although there are reports of CCHFV-positive patients with lower ALP values than CCHFV-negative patients (33, 34). Decreases in ALP concentrations are not often reported during viral infections; however, decreased ALP after periods of fasting has been reported in rats (19).

4′-FlU has been reported to be highly effective against other RNA viruses and potently protects animals against challenge, a few of which also belong to the *Bunyaviricetes* class (13–17). Remarkably, treatment with 4′-FlU was able to protect 100% and 50% of HAZV-infected animals even when the onset of treatment was delayed until 4 and 5 days p.i., respectively. Notably, mice had already lost 18%–24% of their initial weight when treatment regimens began 4 or 5 days after infection, after which their weights stabilized, and the mice recovered following the initiation of treatment. While our work is the first to report true therapeutic efficacy, initiating therapy after the onset of clear signs of disease, a previous study showed that infection in mice by the related Wetland Virus can be prophylactically treated with 4′-FlU (35). Given its robust efficacy against these orthonairoviruses in their respective mouse models, studies in mouse and non-human primate models to investigate the protective effect of 4′-FlU will be forthcoming.

Finally, our study has its limitations. First, HAZV is a proposed surrogate virus with reports of different entry mechanisms than CCHFV (28). Thus, HAZV infection may not mimic the functional characteristics of CCHFV infection and pathogenesis. Additionally, we cannot rule out the contribution of HAZV present in the blood to our quantification of tissue viral loads, as mice were not perfused before organ collection. This, however, is consistent with the approach used to characterize the CCHFV mouse models (7, 9, 36). Another limitation is the requirement of immunocompromised mice lacking intact type I IFN signaling. However, immunocompromised mouse models are generally more rigorous for testing the efficacy of direct-acting antivirals against CCHFV compared to MA-CCHFV infection in immunocompetent mice (21, 37). Despite its shortcomings, the accessibility of the HAZV mouse model and its similarities with CCHFV infection and disease in rodents, macaques, and humans make it a useful tool to study orthonairovirus pathogenesis and early-stage countermeasure development.

## MATERIALS AND METHODS

### Viruses, cells, and animals

The JC280 strain of HAZV was kindly provided by the World Reference Center for Emerging Viruses and Arboviruses (WRCEVA; University of Texas Medical Branch). The stock used was prepared from two passages in Vero E6 African green monkey kidney cells (American Type Culture Collection; ATCC). Male and female *Ifnar^−/−^* mice on the C57BL/6 background were obtained from the breeding colony at Utah State University.

### Characterization of HAZV disease in *Ifnar*^−/−^ mice

Mice were weighed 3 days before the infection and sorted into cohorts (*n* = 6) to minimize weight and sex differences across all sacrifice groups. They were infected SC with 100 CCID_50_ HAZV or sham-infected with the Minimal Essential Medium (MEM; Cytiva) vehicle. Cohorts of mice were scheduled for sacrifice on days 1–5 p.i. for blood and tissue collection to assess hematology, blood chemistry, cytokine profiling, viral titers, and histopathology. Sham-infected animals (*n* = 1 per day, total *n* = 5) were sacrificed on days 1–5 and included as normal controls for all the analyses.

Anticoagulated whole-blood samples in EDTA were obtained by submandibular bleed and analyzed using an automated CBC instrument (Sysmex XN-1000V; Sysmex). Additional whole blood was processed for serum, and following euthanasia, tissue samples were collected and homogenized in a fixed volume of MEM. Cytokine concentrations in the serum and tissue homogenates were analyzed using the Q-Plex Mouse Cytokine Screen HS (15-plex) enzyme-linked immunosorbent assay (Quansys Biosciences) per the manufacturer’s specifications.

Virus titers were determined using an infectious cell culture assay. Tissue homogenates and serum were serially diluted and added to triplicate wells of Vero E6 cell monolayers in 96-well microplates. Viral cytopathic effect was determined 7 days post-sampling, and the 50% endpoints were calculated using the Reed-Muench method (38). The lower limit of detection for serum samples was 2.45 log_10_ CCID_50_ per mL. The lower limit of detection for tissues was 3.05 log^10^ CCID_50_/g or 4.05 log^10^ CCID_50_/g.

Liver, spleen, kidney, heart, lung, brain, lymph nodes, and thymus harvested from the HAZV-infected mice and five sham-infected controls were fixed in 10% neutral-buffered formalin and processed at the Utah Veterinary Diagnostic Laboratory (UVDL, Logan, UT). Histopathology was performed in a blinded fashion by a board-certified veterinary pathologist. The formalin-fixed tissues were paraffin-embedded (FFPE), sectioned, stained with hematoxylin and eosin (H&E) following routine procedures, and examined by light microscopy. Necrosis severity was subjectively scored as 0 = no lesions, 1 = minimal, 2 = mild, 3 = moderate, and 4 = severe.

To determine the virus tropism and tissue distribution in HAZV-infected *Ifnar^−/−^* mice, ISH was performed on FFPE tissues as described for CCHFV (39). In brief, tissue sections were deparaffinized with xylene, washed with absolute ethanol, and blocked with peroxidase, followed by antigen retrieval for 15 min. The slides were then incubated with the V-HAZV-segmentM-GPC-sense-C1 probe (Advanced Cell Diagnostics; cat.# 1244321-C1), which targets the GPC gene of the JC280 strain of HAZV, at 40°C for 2 h. To visualize HAZV RNA in the various tissues, the RNAscope 2.5 High Definition-Red Assay kit (Advanced Cell Diagnostics; cat.# 322350) was used following the manufacturer’s instructions (40). Briefly, the slides were washed twice for 2 min with phosphate-buffered saline, followed by incubation with a total of 6 amplifier probes conjugated to alkaline phosphatase. The signal was detected using a coupled naphthol phosphate and diazonium salt substrate (Fast Red dye) at room temperature for 10 min and counterstained with hematoxylin.

### Inhibition of HAZV infection in cell culture by 4′-FlU

To assess the inhibitory activity of 4′-FlU against HAZV infection in Vero E6 cells, a virus yield reduction assay was used. Vero E6 cells seeded in 96-well microplates were infected with HAZV at a multiplicity of infection of 0.1 and treated with half-log_10_ dilutions of 4′-FlU (Emory Institute for Drug Development) in MEM supplemented with 2% fetal bovine serum (FBS; Sigma-Aldrich). The antiviral drug favipiravir (TargetMol) was included for comparison. The culture supernatants were collected from the plates after incubation for 4 days to assess HAZV titers. After thawing, the supernatants were titrated for infectious virus by endpoint dilution. The EC_90_ was determined by regression analysis and represents the drug concentration that reduced the virus yield by one log_10_ unit.

### 4′-FlU treatment of HAZV infection in *Ifnar*^−/−^ mice

#### Prophylactic efficacy study

Three days prior to infection, 6- to 9-week-old *Ifnar^−/−^* mice were weighed and assigned to groups so that sex and weight were evenly distributed. Groups of mice (*n* = 14) were treated PO, QD for 8 days with 15, 5, or 1.5 mg/kg 4′-FlU or an equal volume of vehicle placebo beginning 2 h before HAZV challenge (SC injection of 0.2 mL) with an approximate challenge dose of 20 CCID_50_. Each of the dosing concentrations for 4′-FlU was prepared daily in 10 mM trisodium citrate (Amresco) in sterile water. Favipiravir, dosed at 200 mg/kg/day, was dissolved in a 21.7 mg/mL meglumine solution and administered IP, twice daily, for 8 days as the positive control treatment. Four animals from each treatment group and the placebo group were preselected for sacrifice on day 6 p.i. to determine liver, spleen, and serum viral titers. The remaining animals were observed for 22 days for morbidity and mortality, and daily body weights were recorded.

#### Therapeutic efficacy study

Three days before infection, 6- to 8-week-old *Ifnar^−/−^* mice were weighed and assigned to groups so that sex and weight were evenly distributed. Cohorts of mice (*n* = 10–14) were treated with 10 mg/kg 4′-FlU or an equal volume of the vehicle placebo treatment beginning 2, 3, 4, or 5 days p.i. or 2 h prior to HAZV challenge with 100 CCID_50_ of virus by SC injection in a 0.2 mL volume. Four animals from each treatment group (except day 5) and the placebo group were preselected for sacrifice on day 5 p.i. to assess viremia and tissue viral loads (spleen, liver). The remaining animals were observed for 21 days for morbidity and mortality, and daily body weights were recorded.

### Statistical analysis

The log-rank (Mantel-Cox) test was used to analyze Kaplan-Meier survival plots. A one-way analysis of variance with Dunnett’s post-test to correct for multiple comparisons was used to compare differences in viral titers, weight change, cytokine concentrations, hematology parameters, and biochemical markers. All statistical evaluations were conducted using Prism 10 (GraphPad Software).

## Data Availability

All data that support the findings of this study are included within this paper and its Supplemental Information file.

## SUPPLEMENTAL MATERIAL

Supplemental material jvi.01421-25-s0001.docx Tables S1 and S2; Fig. S1 and S2.

ASM does not own the copyrights to Supplemental Material that may be linked to, or accessed through, an article. The authors have granted ASM a non-exclusive, world-wide license to publish the Supplemental Material files. Please contact the corresponding author directly for reuse.
