# Supplementary material for: Pathogenesis of Hazara orthonairovirus infection in type I interferon receptor-deficient mice and resolution of disease following 4′-fluorouridine therapy
Source: J Virol. 2025 Nov 18;99(12):e01421-25. doi: 10.1128/jvi.01421-25 (PMC12724280; doi:10.1128/jvi.01421-25)
Supplement: Supplemental material — Tables S1 and S2; Fig. S1 and S2. [file jvi.01421-25-s0001.docx]

**Supplemental Table 1. Cytokine response to HAZV infection in *Ifnar^-/-^* mice.**

| **Cytokine** | **Tissue** | **Day 1** | **Day 2** | **Day 3** | **Day 4** | **Day 5** | **Control** |
| --- | --- | --- | --- | --- | --- | --- | --- |
| **IL-1α** | Serum^a^ | 41 ± 33 | 218 ± 235 | 127 ± 83 | 150 ± 115 | 148 ± 183 | 82 ± 48 |
|  | Spleen^b^ | 4244 ± 1761 | 6351 ± 1334 | 16346 ± 2400*** | 13450 ± 1545*** | 12365 ± 2032*** | 5447 ± 1838 |
|  | Liver^b^ | 697 ± 413 | 1004 ± 793 | 3617 ± 1571*** | 4233 ± 345*** | 4511 ± 880*** | 1141 ± 214 |
| **IL-1β** | Serum | 33 ± 7.8 | 35 ± 5.3 | 42 ± 14 | 40 ± 11 | 41 ± 25 | 34 ± 2.9 |
|  | Spleen | 1945 ± 1365 | 2007 ± 1651 | 10862 ± 6338** | 5708 ± 2628 | 2566 ± 902 | 2759 ± 2708 |
|  | Liver | 1735 ± 357 | 1276 ± 250 | 5115 ± 1830*** | 3764 ± 291* | 4473 ± 641*** | 2011 ± 751 |
| **IL-2** | Serum | 2.5 ± 0 | 2.7 ± 0.53 | 2.6 ± 0.15 | 3.0 ± 0.15 | 3.9 ± 0.65 | 2.7 ± 0.42 |
|  | Spleen | 37 ± 18 | 59 ± 14 | 65 ± 14 | 52 ± 9.0 | 36 ± 6.3 | 48 ± 24 |
|  | Liver | 228 ± 51 | 150 ± 31 | 340 ± 148 | 353 ± 39 | 552 ± 84*** | 259 ± 102 |
| **IL-3** | Serum | 0.36 ± 0 | 0.37 ± 0.02 | 0.40 ± 0.08 | 0.38 ± 0.06 | 0.38 ± 0.60 | 0.43 ± 0.15 |
|  | Spleen | 2.8 ± 1.0 | 5.4 ± 1.8 | 6.0 ± 1.6 | 22 ± 11*** | 8.4 ± 3.1 | 4.3 ± 1.6 |
|  | Liver | 28 ± 4.9 | 15 ± 3.3** | 45 ± 9.0* | 37 ± 4.6 | 53 ± 9.2*** | 30 ± 11.7 |
| **IL-4** | Serum | 2.2 ± 0 | 2.2 ± 0 | 2.2 ± 0.1 | 2.2 ± 0 | 2.2 ± 0 | 2.2 ± 0 |
|  | Spleen | 29 ± 8.6 | 54 ± 17 | 78 ± 15** | 47 ± 13 | 36 ± 7.7 | 45 ± 21 |
|  | Liver | 200 ± 47 | 129 ± 29 | 331 ± 153 | 320 ± 37 | 524 ± 100*** | 238 ± 109 |
| **IL-5** | Serum | 17 ± 12 | 54 ± 55 | 69 ± 61 | 128 ± 116 | 120 ± 90 | 45 ± 78 |
|  | Spleen | 220 ± 51 | 421 ± 102 | 459 ± 75 | 292 ± 31 | 256 ± 24 | 349 ± 231 |
|  | Liver | 1334 ± 247 | 985 ± 181 | 1893 ± 680 | 2074 ± 345 | 2916 ± 439*** | 1530 ± 517 |
| **IL-6** | Serum | 43 ± 40 | 37 ± 44 | 1391 ± 759 | 5377 ± 3531*** | 2867 ± 1499* | 25 ± 21 |
|  | Spleen | 165 ± 75 | 292 ± 91 | 2376 ± 2269 | 2204 ± 2461 | 518 ± 174 | 277 ± 145 |
|  | Liver | 867 ± 301 | 512 ± 144 | 1366 ± 384 | 2913 ± 763*** | 1844 ± 167*** | 762 ± 263 |
| **IL-10** | Serum | 7.1 ± 0 | 7.1 ± 0 | 7.3 ± 0.36 | 37 ± 21* | 73 ± 30*** | 7.2 ± 0.25 |
|  | Spleen | 59 ± 33 | 97 ± 28 | 110 ± 32 | 85 ± 12 | 92 ± 15 | 75 ± 23 |
|  | Liver | 444 ± 100 | 305 ± 69 | 665 ± 275 | 707 ± 76 | 1075 ± 177*** | 527 ± 213 |
| **IL-12p70** | Serum | 7.4 ± 0 | 7.4 ± 0 | 7.6 ± 0.36 | 17 ± 8.8 | 24 ± 24 | 7.5 ± 0.15 |
|  | Spleen | 35 ± 4.7* | 72 ± 21 | 75 ± 23 | 50 ± 8.4 | 46 ± 8.8 | 67 ± 22 |
|  | Liver | 340 ± 95 | 212 ± 63 | 537 ± 252 | 565 ± 84 | 963 ± 196*** | 422 ± 204 |
| **IL-17A** | Serum | 8.9 ± 0 | 9.9 ± 2.5 | 9.5 ± 1.4 | 9.7 ± 1.8 | 15 ± 12 | 10 ± 3.0 |
|  | Spleen | 61 ± 22 | 118 ± 48 | 128 ± 41 | 76 ± 16 | 63 ± 15 | 100 ± 32 |
|  | Liver | 471 ± 107 | 311 ± 68 | 701 ± 308 | 731 ± 88 | 1155 ± 215*** | 562 ± 228 |
| **IFN-γ** | Serum | 11 ± 0 | 11 ± 0 | 188 ± 183 | 865 ± 562*** | 507 ± 409* | 11 ± 0 |
|  | Spleen | 82 ± 29 | 176 ± 48 | 298 ± 97 | 1221 ± 538*** | 1164 ± 617*** | 171 ± 47 |
|  | Liver | 604 ± 166 | 386 ± 103 | 1051 ± 426 | 1126 ± 180 | 1719 ± 338*** | 742 ± 328 |
| **TNF-α** | Serum | 5.3 ± 0 | 6.1 ± 2.0 | 181 ± 105*** | 278 ± 85*** | 252 ± 39*** | 5.4 ± 0.13 |
|  | Spleen | 70 ± 25 | 103 ± 14 | 628 ± 365** | 742 ± 297*** | 398 ± 125 | 92 ± 42 |
|  | Liver | 196 ± 43 | 138 ± 28 | 449 ± 158** | 643 ± 135*** | 677 ± 114*** | 235 ± 89 |
| **MIP-1α** | Serum | 8.2 ± 0 | 8.2 ± 0 | 28 ± 21 | 43 ± 42* | 23 ± 20 | 8.2 ± 0 |
|  | Spleen | 214 ± 40 | 299 ± 42 | 5856 ± 3995** | 6494 ± 2817*** | 3970 ± 1174* | 232 ± 142 |
|  | Liver | 322 ± 80 | 208 ± 54 | 2508 ± 1180*** | 3159 ± 846*** | 3042 ± 771*** | 382 ± 117 |
| **GM-CSF** | Serum | 5.5 ± 0 | 6.2 ± 1.8 | 5.7 ± 0.36 | 5.5 ± 0 | 9.9 ± 9.6 | 6.2 ± 1.4 |
|  | Spleen | 88 ± 25 | 165 ± 49 | 182 ± 45 | 144 ± 66 | 105 ± 25 | 126 ± 57 |
|  | Liver | 517 ± 103 | 373 ± 58 | 723 ± 265 | 758 ± 67 | 1111 ± 162*** | 576 ± 187 |
| **RANTES** | Serum | 32 ± 8.7 | 37 ± 15 | 372 ± 228 | 7898 ± 5107*** | 4020 ± 2059* | 39 ± 22 |
|  | Spleen | 5052 ± 2385 | 8180 ± 3236 | 10057 ± 2659 | 69117 ± 17036*** | 61939 ± 11700*** | 10089 ± 7001 |
|  | Liver | 1106 ± 269 | 744 ± 153 | 1856 ± 952 | 16842 ± 9008*** | 19529 ± 9503*** | 1312 ± 605 |

^a^Serum values are expressed as pg/mL of serum ± standard deviation.

^b^Tissue values are reported as pg/g of tissue ± standard deviation.

Data from infected mice are from 6 animals per day. ****p* < 0.001, ***p* < 0.01, **p* < 0.05 compared to sham-infected normal controls (*n* = 5 for serum and liver; *n* = 4 for spleen). Significant differences are indicated in red font. In samples with an undetectable concentration of analyte, a value representative of the lower limit of detection (highlighted in gray) was assigned.

**Supplemental Table 2. Summary of hematologic findings in HAZV-challenged *Ifnar^-/-^ mice***

| **Parameter^a^** | **Day 1^b^** | **Day 2^b^** | **Day 3** | **Day 4** | **Day 5** | **Controls^b^** |
| --- | --- | --- | --- | --- | --- | --- |
| RBC | 11.9 ± 1.5 | 10.4 ± 0.6 | 11.3 ± 0.2 | 11.0 ± 0.3 | 10.8 ± 0.5 | 11.6 ± 0.5 |
| HGB | 18.4 ± 2.4 | 16.5 ± 1.1 | 16.9 ± 0.2 | 16.2 ± 0.7 | 15.8 ± 0.7* | 17.5 ± 1.0 |
| HCT | 53.3 ± 7.0 | 47.7 ± 3.7 | 51.5 ± 0.9 | 48.0 ± 1.3 | 46.6 ± 1.9* | 51.7 ± 2.2 |
| RDW-CV | 14.7 ± 0.8 | 15.4 ± 1.1 | 19.1 ± 0.2 | 18.8 ± 0.9 | 18.0 ± 0.4 | 16.2 ± 3.3 |
| NRBC# | 0.05 ± 0.03 | 0.04 ± 0.01 | 0.07 ± 0.07 | 0.09 ± 0.07 | 0.02 ± 0.01 | 0.07 ± 0.02 |
| MCV | 44.9 ± 0.1 | 45.7 ± 1.1 | 45.7 ± 0.6 | 43.8 ± 0.2 | 43.1 ± 0.3** | 44.6 ± 0.8 |
| MCH | 15.5 ± 0.0 | 15.8 ± 0.2 | 15.0 ± 0.3 | 14.8 ± 0.3 | 14.6 ± 0.1 | 15.1 ± 0.4 |
| MCHC | 34.6 ± 0.1 | 34.6 ± 1.1 | 32.9 ± 0.5 | 33.8 ± 0.6 | 33.9 ± 0.2 | 33.7 ± 0.6 |
| PCT | 0.73 ± 0.02 | 0.89 ± 0.11 | 0.64 ± 0.19 | 0.18 ± 0.12*** | 0.24 ± 0.12*** | 0.81 ± 0.03 |
| PLT | 1035 ± 27 | 1259 ± 195 | 950 ± 394 | 201 ± 147*** | 229 ± 133*** | 1191 ± 30 |
| MPV | 7.0 ± 0.07 | 7.1 ± 0.39 | 6.4 ± 0.18 | 7.3 ± 0.49 | 7.7 ± 0.50** | 6.7 ± 0.46 |
| PDW | 5.6 ± 0.28 | 5.4 ± 0.19 | 6.7 ± 0.19 | ___^c^ | 9.4^c^ | 6.2 ± 0.65 |
| WBC | 7.5 ± 0.35 | 9.2 ± 2.1 | 3.5 ± 3.0** | 2.1 ± 0.67*** | 2.7 ± 0.45*** | 7.7 ± 0.80 |
| Neut# | 1.2 ± 0.48 | 2.0 ± 1.94 | 1.3 ± 0.76 | 1.0 ± 0.53 | 1.3 ± 0.48 | 0.82 ± 0.30 |
| Lymph# | 5.9 ± 0.74 | 6.4 ± 1.4 | 1.9 ± 2.2*** | 0.86 ± 0.41*** | 0.99 ± 0.53 *** | 6.4 ± 0.68 |
| Mono# | 0.21 ± 0.04 | 0.43 ± 0.10 | 0.16 ± 0.21 | 0.17 ± 0.07 | 0.33 ± 0.15 | 0.26 ± 0.11 |
| Eos# | 0.14 ± 0.06 | 0.42 ± 0.67 | 0.08 ± 0.10 | 0.02 ± 0.01 | 0.02 ± 0.01 | 0.20 ± 0.09 |
| Baso# | 0.02 ± 0.00 | 0.02 ± 0.01 | 0.01 ± 0.01 | 0.03 ± 0.01 | 0.03 ± 0.01 | 0.02 ± 0.02 |
| Neut% | 15.8 ± 7.1 | 19.4 ± 13.6 | 40.4 ± 18.5* | 47.0 ± 14.9** | 49.6 ± 14.8** | 10.6 ± 3.3 |
| Lymph% | 79.3 ± 6.1 | 70.4 ± 14.2 | 49.3 ± 12.2** | 42.4 ± 14.7*** | 35.6 ± 16.2*** | 83.2 ± 3.8 |
| Mono% | 2.8 ± 0.42 | 4.8 ± 1.1 | 4.2 ± 2.1 | 8.2 ± 2.7 | 13.1 ± 7.7** | 3.3 ± 1.3 |
| Eos% | 1.9 ± 0.64 | 5.2 ± 8.9 | 5.6 ± 11.2 | 0.87 ± 0.44 | 0.78 ± 0.52 | 2.6 ± 1.1 |
| Baso% | 0.30 ± 0.00 | 0.18 ± 0.08 | 0.58 ± 0.49 | 1.5 ± 0.77** | 0.92 ± 0.34 | 0.23 ± 0.21 |

^a^Data are reported as the group means and standard deviation or spread. ****p* < 0.001, ***p* < 0.01, **p* < 0.05 compared to normal controls (*n* = 4). Significant differences are indicated in red font.

^b^Inadequate blood samples were obtained from 4 animals in the day 1 group, 1 from day 2, and 1 of the control animals for CBC analysis.

^c^PDW values could not be obtained from all the blood samples taken on day 4 p.i and all but one sample on day 5 p.i.

RBC, red blood cells; HGB, hemoglobin; HCT, hematocrit; RDW-CV, red cell distribution width - coefficient of variation; NRBC#, nucleated red blood cell number; MCV, mean corpuscular volume; MCH, mean corpuscular hemoglobin; MCHC, mean corpuscular hemoglobin concentration; PCT, plateletcrit; PLT, platelets; MPV, mean platelet volume; PDW, platelet distribution width; WBC, white blood cells; Neut, neutrophils; Lymph, lymphocytes; Mono, monocytes; Eos, eosinophils; Baso, basophils.

**Supplemental Figure 1. Histological lesions and in situ localization of HAZV in tissues of mice with advanced disease.** Histology (left panels) of *Ifnar^-/-^* mice infected with HAZV and in situ hybridization (ISH; right panels) for HAZV RNA at day 4 p.i. No lesions are detected in the A) brain, E) heart, F) lung, and M) kidney of sham-infected mice. No lesions are detected in the B) brain or N) kidney of infected mice. F) Neutrophilic myocarditis and epicarditis and J) pulmonary thrombi are detected in the heart and lung of infected mice. No viral RNA is detected in the C) brain, G) heart, K) lung, or O) kidney of sham-infected mice. Viral RNA is present in endothelial cells and intravascular cells with a morphology consistent with monocytes in the D) brain, H) heart, L) lung, and P) kidney of infected mice. H&E stain (A, B, E, F, I, J, M, N). ISH with hematoxylin counterstain (C, D, G, H, K, L, O, P). 200× magnification. Bar = 100 μm.


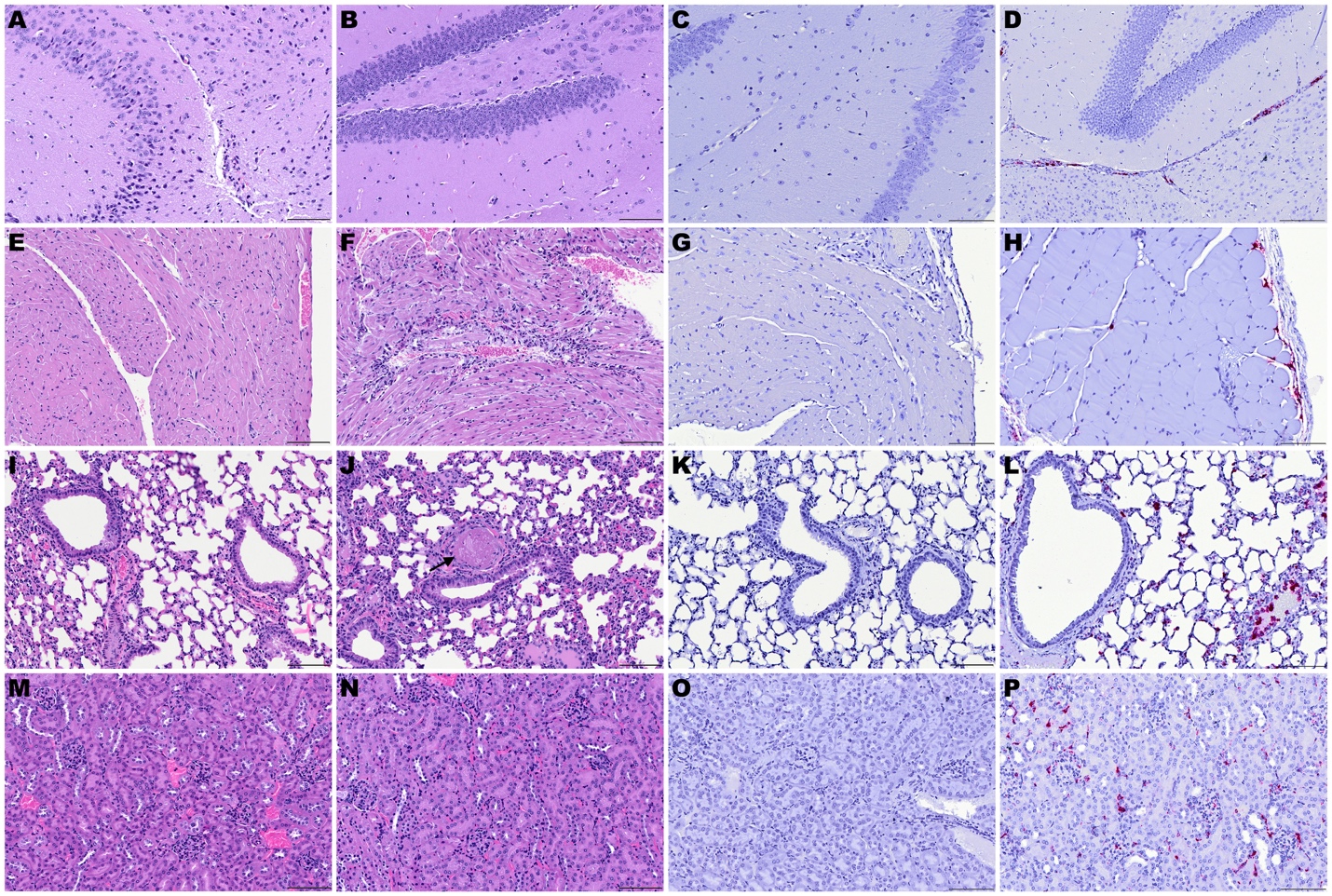


**Supplemental Figure 2. Prophylactic 4’-FlU treatment protects *Ifnar^-/-^* mice against HAZV infection and disease.** A) Survival of animals challenged with HAZV and treated with either 15, 5, or 1.5 mg/kg/day of 4’-FlU, 200 mg/kg/day of favipiravir, or with vehicle placebo (*n* = 10/group). Sham-infected normal controls (*n* = 3/group) were included for comparison. ***p* < 0.01, compared to the vehicle placebo control group. B) Daily body weight data are represented as the group mean and standard error of the percent change in weight of animals relative to their starting weights on day 0, the day of virus challenge. C) Day 6 p.i. percent weight change of individual animals from the survival (*n* = 10/group) and day 6 p.i. sacrifice (*n* = 4) cohorts (total *n* = 14). *****p* < 0.0001, compared to the vehicle placebo control group. D) Subsets of animals in each group (*n* = 4) were predesignated for sacrifice on day 6 p.i. to assess liver, spleen, and serum virus titers. One animal in the placebo group succumbed the night before sacrifice and, therefore, is not included in the analysis. The dashed (serum) and dotted (liver, spleen) lines represent the assay lower limit of detection.
